# 稳定表达荧光素酶的nm23-H1表达缺失人肺腺癌细胞株的构建及其体内外活性检测

**DOI:** 10.3779/j.issn.1009-3419.2012.03.04

**Published:** 2012-03-20

**Authors:** 红明 王, 大兴 朱, 志浩 吴, 清华 周

**Affiliations:** 300052 天津，天津市肺癌转移与肿瘤微环境重点实验室，天津市肺癌研究所，天津医科大学总医院 Tianjin Key Laboratory of Lung Cancer Metastasis and Tumor Microenvironment, Tianjin Lung Cancer Institute, Tianjin Medical University General Hospital, Tianjin 300052, China

**Keywords:** 荧光素酶, 肺腺癌, 生物发光成像, nm23-H1, Luciferase, Lung adenocarcinoma, Bioluminescence imaging, nm23-H1

## Abstract

**背景与目的:**

在实验动物存活条件下，通过活体成像技术能探测到标记有萤火虫荧光素酶（*luc*）基因的肿瘤细胞在体内的分布情况。本研究旨在稳定表达nm23-H1 shRNA的人肺腺癌细胞株A549中，建立能稳定表达萤火虫荧光素酶的发光细胞株A549/nm23-H1-shRNA-luc，并检测其体内外发光情况，为下一步相关的体内实验提供实验材料。

**方法:**

通过浓度梯度法测定A549/nm23-H1-shRNA细胞的潮霉素最佳筛选浓度，将带有萤火虫荧光素酶基因的PGL4.50质粒转入A549/nm23-H1-shRNA细胞中，利用潮霉素筛选单克隆细胞株A549/nm23-H1-shRNA-luc，并采用生物发光技术对单克隆细胞株进行阳性鉴定并挑选发光最强的1个克隆分析其表达荧光素酶的稳定性。为检测A549/nm23-H1-shRNA-luc细胞在体内发光的稳定性，将A549/nm23-H1-shRNA-luc细胞接种于10只裸鼠右后腹股沟皮下之后，并随机分为两组，每组5只裸鼠，运用活体成像系统观察成像情况。

**结果:**

A549/nm23-H1-shRNA-luc细胞的潮霉素最佳筛选浓度为300 μg/mL。经过潮霉素筛选所建立的A549/nm23-H1-shRNA-luc细胞株能在体外稳定表达荧光素酶，细胞数（x）和生物发光值（y）呈直线相关，回归方程是：y=3, 699.9x+992, 237，*R^2^*=0.975, 1。为评估此细胞株在体内生物发光的稳定性，将细胞种植进入10只裸鼠并随机分成两组，结果显示体内生物发光值差异不具有统计学意义（*P* > 0.05）。

**结论:**

成功建立了能持续、稳定表达荧光素酶的A549/nm23-H1-shRNA-luc细胞株。

## Abstract (en)

**Background and objective:**

On the condition that laboratory animals survive, we can detect the distribution of tumor cells by *in vivo* imaging that were labeled with firefly- luciferase (*luc*) gene. The purpose of this study is to establish a light-emitting cell line A549/nm23-H1-shRNA-luc which could express nm23-H1 shRNA and firefly-luciferase stably, and detect its bioluminescence *in vitro* and *in vivo*. It will provide the preparation for the next related experimental research *in vivo*.

**Methods:**

The optimal concentration of hygromycin B for screening A549/nm23-H1-shRNA cells was determined by concentration gradient method. We firstly transfected the plasmid (PGL4.50) with luc gene into A549/nm23-H1-shRNA cells and then screened the monoclonal cell line A549/nm23-H1-shRNA-luc with hyhromycin B. The positive monoclonal cell line was identified with an *in vivo* imaging system, thereafter the expression stability of luciferase was analyzed in the strongest light-emitting positive monoclonal cell line. The A549/nm23-H1-shRNA-luc cells were inoculated subcutaneously into right-hind groin of nude mice and then observed by the *in vivo* imaging system.

**Results:**

The optimal concentration of hygromycin B used in screening A549/nm23-H1-shRNA cells was 300 μg/mL. After screening, the A549/nm23-H1-shRNA-luc cells established can express luciferase stably *in vitro*, a great linear correlation existed between the amount of cells (x) and bioluminescence values (y), with an equation of y=3, 699.9x+992, 237, and the square of the correlation coefficient (*R^2^*) was 0.975, 1. To evaluate the stability of bioluminescence *in vivo*, 10 nude mice were randomly divided into two groups that the same number of cells were implanted into. The variation of bioluminescence values detected *in vivo* between the two groups of the same cells was not statistically significant (*P* > 0.05).

**Conclusion:**

We have successfully established the cell line A549/nm23-H1-shRNA-luc which can express luciferase persistently and stably.

癌症极大地威胁着人类的健康，其中肺癌占据全球恶性肿瘤死亡原因的首位^[1]^。肺腺癌是肺癌的重要组织学类型，其恶性程度很高，极易出现浸润和转移。肺癌晚期的主要表现是发生转移，它是肺癌的恶性标志和特征，也是肺癌患者治疗失败和死亡的主要原因，*nm23-H1*基因的低表达就是肺癌细胞转移的重要原因之一^[2, 3]^。近年来，随着活体生物发光技术被广泛应用于动物模型的研究，我们能够通过活体生物成像系统直接监控生物体内的细胞活动和基因行为。特别是在各种类型的肿瘤研究中，能够直接快速地测量各种肿瘤动物模型中肿瘤细胞的生长和转移状态，并可对治疗中肿瘤细胞的变化进行实时观测和评估。能够无创伤地对动物整体的原位瘤、转移瘤及自发瘤进行检测及计量，即使微小的迁移也能被检出^[4-6]^。

本实验室先前已成功构建nm23-H1表达缺失的人肺腺癌细胞株A549/nm23-H1-shRNA。在此基础上，本研究通过稳定转染携带有萤火虫荧光素酶（*luc*）基因的质粒进入该细胞株中，利用潮霉素B筛选出能稳定表达荧光素酶的细胞株A549/nm23-H1-shRNA-luc。借助活体成像技术，对构建的细胞株进行体内外生物发光检测，为后续转移相关的动物试验的研究奠定好基础。

## 1 材料与方法

### 1.1 材料

nm23-H1表达缺失的人肺腺癌细胞株A549（A549/nm23-H1-shRNA）由本实验室构建；细胞培养基是RPMI-1640（GIBCO）并添加10%的小牛血清（NCS, GIBCO）；质粒表达载体（PGL4.50）购自Promega Corporation；质粒大提取盒购自Qiagen公司（Hispeed Plasmid Maxi Kit）；感受态细胞（Trans5α Chemically Competent Cell）购自北京全式金生物技术有限公司；转染试剂为PolyJet^TM^ *In Vitro* DNA Transfection Reagent（SignaGen Laboratories）；筛选试剂为潮霉素B购自德国Merck公司；荧光素（Luciferin）购自Promega Corporation；实验动物为裸鼠，雌性，4周龄，15 g左右，购自北京维通利华实验动物技术有限公司。精诺真活体成像系统（购自美国公司，IVIS200）由本实验室提供。

### 1.2 方法

#### 1.2.1 质粒扩增与提取

按常用方法进行细菌转化和质粒扩增。质粒提取过程参照Qiagen公司提供的大提取试剂盒说明书进行。所提取的质粒利用紫外分光光度计（Beckman DU800）进行纯度和浓度的测定。

#### 1.2.2 潮霉素最佳筛选浓度的测定

将处于对数生长期的nm23-H1表达缺失人肺腺癌细胞株A549/nm23-H1-shRNA用0.25%胰酶消化，以5×10^4^个/孔接种于24孔板中，加入含10%NCS的RPMI-1640培养基于37 ℃、5%CO_2_的培养箱中培养过夜。铺板第2天加入不同浓度的潮霉素（50 μg/mL、100 μg/mL、150 μg/mL、200 μg/mL、250 μg/mL、300 μg/mL、350 μg/mL、400 μg/mL、450 μg/mL、500 μg/mL、550 μg/mL、600 μg/mL），每3天换液1次，换液的同时加入以上不同浓度的潮霉素，共培养2周-3周。

#### 1.2.3 细胞转染

##### 1.2.3.1 A549/nm23-H1-shRNA人肺腺癌细胞的培养

转染前1天，用0.25%胰酶消化细胞，利用含10%NCS的RPMI-1640培养基将处于对数生长期的细胞制备成单细胞悬液，按3×10^5^个/孔接种于6孔板中，每孔培养基为2 mL，置于37 ℃、5%CO_2_培养箱中培养，待细胞生长至铺满孔底80%左右后进行试验。

##### 1.2.3.2 PolyJet^TM^/DNA混合液的制备及转染

转染当天每个培养孔更换为无小牛血清的RPMI-1640培养基1 mL。取6 μg PGL4.50质粒与300 μL无血清含高糖的DMEM培养基轻轻混合均匀，标记为A管，再将18 μL PolyJet^TM^与300 μL无血清含高糖的DMEM培养基轻轻混合均匀，标记为B管，然后立即将B管中转染试剂混合液加入A管中充分混合。室温孵育15 min后将PolyJet^TM^/DNA悬液按100 μL/孔缓缓加入6孔板中，轻轻摇匀后置于37 ℃、5%CO_2_培养箱中继续培养，5 h后，更换新鲜的含10%NCS的RPMI-1640培养基。

##### 1.2.3.3 药物筛选

转染48 h后，弃去培养基，用PBS洗1遍。加入含有潮霉素的培养基进行筛选。每2-4天观察细胞生长情况，并重新更换含有潮霉素的培养基。2周后，出现克隆。

##### 1.2.3.4 筛选稳定的细胞株

挑选生长状态良好的单克隆至96孔板中，用含潮霉素的培养基继续培养，待细胞约90%-100%汇合后，转移至48孔板继续培养。同样，当细胞生长至90%-100%左右时扩至24孔板中培养，再12孔板，6孔板，最后至90 mm的培养皿中培养。取部分细胞用0.25%的胰酶充分消化后，用PBS洗涤2遍，于IVIS系统成像观察，挑选发光最强的1个克隆，分别冻存、继续扩增培养。

##### 1.2.3.5 A549/nm23-H1-shRNA-luc细胞体外成像及其稳定性评估

将阳性单克隆细胞株用0.25%胰酶消化、计数，取1×10^4^个细胞悬浮于100 μL培养基中，按1:2的比例逐孔稀释，接种于黑色的96孔板中，使细胞数按2:1比例逐孔递减，分别加入荧光素（150 μg/mL），另设2孔作为阴性对照。静置3 min-4 min后，成像1 min。生物发光值按每秒光子量（photons/s）计算。继续将A549/nm23-H1-shRNA-luc细胞持续培养9周（共计40代），每隔8代利用活体成像技术检测细胞生物发光情况一次，以观察*luc*基因是否稳定表达。

##### 1.2.3.6 A549/nm23-H1-shRNA-luc细胞在活体内发光影像及其稳定性的检测

每只裸鼠（共10只，雌性，每只约15 g）右后腹股沟处皮下注射0.1 mL的A549/nm23-H1-shRNA-luc细胞悬液（浓度为4×10^7^个/mL），然后给每只裸鼠腹腔内注射0.2 mL的荧光素（用1×PBS配置成15 mg/mL的荧光素，并过滤）。7 min-8 min后，将其放入麻醉箱内用气体异氟烷麻醉，并成像1 s。观察及测量结束后，断颈处死裸鼠。

### 1.3 统计学处理

采用SPSS 17.0统计软件及Excel 2003分析作图，资料描述使用均值、标准差等指标。统计推断运用方差分析、直线相关等方法，*P* < 0.05为差异具有统计学意义。

## 2 结果

### 2.1 潮霉素最佳筛选浓度的测定结果

按实验设计加入不同浓度的潮霉素（50 μg/mL、100 μg/mL、150 μg/mL、200 μg/mL、250 μg/mL、300 μg/mL、350 μg/mL、400 μg/mL、450 μg/mL、500 μg/mL、550 μg/mL、600 μg/mL），培养5 d后，每孔均可见不同程度的细胞死亡，培养14 d后，潮霉素浓度为300 μg/mL、350 μg/mL及以上的组的细胞均死亡，并可见细胞骨架及碎片。其它组浓度的细胞虽也有不同程度的细胞死亡但未完全死亡。因此可以确定潮霉素对nm23-H1表达缺失的人肺腺癌细胞A549（A549/nm23-H1-shRNA）的最佳筛选浓度为300 μg/mL（图 1）。

**1:**
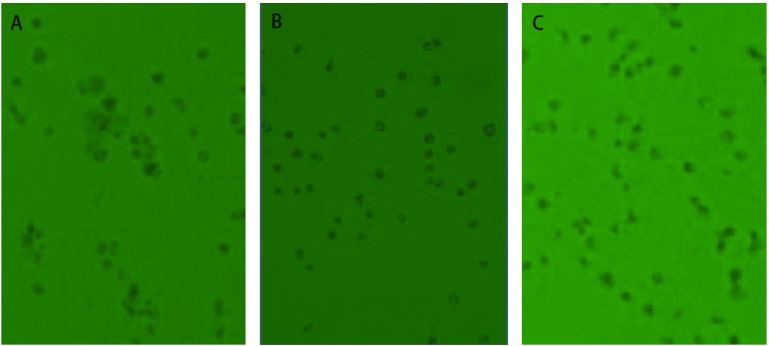
浓度梯度实验中，A549/nm23-H1-shRNA细胞在加不同浓度潮霉素14 d后显微镜下的变化（×40）。A：A549/nm23-H1-shRNA细胞在潮霉素浓度为250 *μ*g/mL下14 d后的变化；B：A549/nm23-H1-shRNA细胞在潮霉素浓度为300 *μ*g/mL下14 d后的变化；C：A549/nm23-H1-shRNA细胞在潮霉素浓度为350 *μ*g/mL下14 d后的变化。 Morphologic changes of A549/nm23-H1-shRNA cells under microscope which were added different doses of hygromycin B in 14 days in concentration gradient experiment (×40). A: The changes of A549/nm23-H1-shRNA cells under microscope which were added hygromycin B (250 *μ*g/mL) in 14 days; B: The changes of A549/nm23-H1-shRNA cells under microscope which were added hygromycin B (300 *μ*g/mL) in 14 days; C: The changes of A549/nm23-H1-shRNA cells under microscope which were added hygromycin B (350 *μ*g/mL) in 14 days.

### 2.2 细胞转染后阳性克隆的形成

A549/nm23-H1-shRNA细胞在80%汇合度时应用PolyJet^TM^转染试剂转染携带有*luc*基因的质粒PGL4.50。48 h后，加入潮霉素（300 μg/mL）。5 d后大部分细胞死亡，仅有少部分细胞存活，并形成克隆（14 d）。将形成的克隆继续在含300 μg/mL潮霉素的培养基中筛选扩增（图 2）。

**2:**
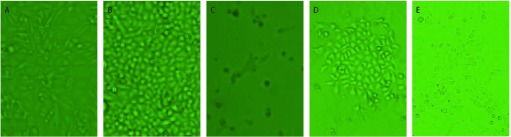
A549/nm23-H1-shRNA细胞转染前后及加潮霉素筛选后显微镜下变化（×40）。A：A549/nm23-H1-shRNA细胞在80%汇合度时的细胞形态；B：A549/nm23-H1-shRNA细胞在转染PGL4.50质粒48 h后的细胞形态；C：A549/nm23-H1-shRNA细胞在转染后加潮霉素筛选5 d后仅存的细胞形态；D：A549/nm23-H1-shRNA细胞在转染后加潮霉素筛选14 d后单克隆的细胞形态；E：A549/nm23-H1-shRNA-luc细胞铺满孔底后的细胞形态。 Morphologies of A549/nm23-H1-shRNA cells before and after being transfected with PGL4.50 and changes after being added hygromycin B (×40). A: The morphology of A549/nm23-H1-shRNA cells up to 80%; B: The morphology of A549/nm23-H1-shRNA cells after 48 h of being transfected with PGL4.50; C: The morphology of A549/nm23-H1-shRNA cells that survived after 5 days of being added hygromycin B; D: The morphology of the monoclonal cells after 14 days of being added hygromycin B; E: The morphology of A549/nm23-H1-shRNA-luc cells up to 100%.

### 2.3 A549/nm23-H1-shRNA-luc细胞体外成像鉴定

携带有*luc*基因的质粒经扩增和纯化后可以有效转染nm23-H1表达缺失的人肺腺癌A549细胞，在含有300 μg/mL潮霉素培养液中可筛选出高表达的阳性细胞株。由图 3可见，每孔最小可探测到的细胞数为39个。细胞发光值（y）对细胞数目（x）的回归方程是：y=3, 699.9x+992, 237，*R^2^*=0.975, 1，显示细胞数目与发光值呈直线相关（图 4），同时也证明了荧光素酶基因已经稳定转入A549/nm23-H1-shRNA细胞中。

**3:**
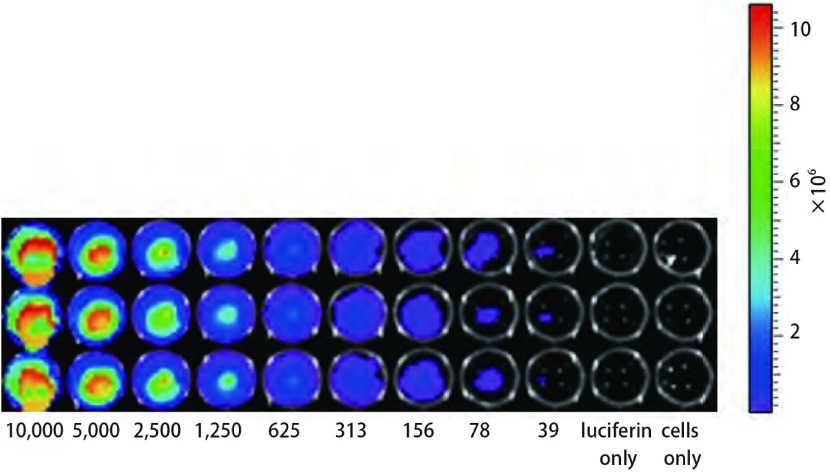
A549/nm23-H1-shRNA-luc细胞活体外成像图示，最小可检测到的细胞数目为39个。 *In vitro* bioluminescence imaging of A549/nm23-H1-shRNA-luc cells, 39 cells are detectable at least.

**4:**
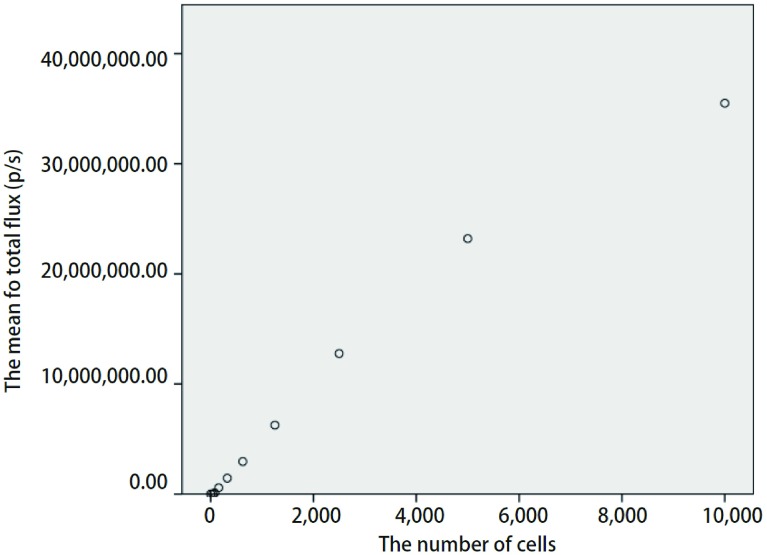
细胞数目和发光值呈明显的直线相关性（*R^2^*=0.975, 1, *P* < 0.001） An apparent correlation between the number of cells and bioluminescence values is well analyzed (*R^2^*=0.975, 1, *P* < 0.001)

### 2.4 评估A549/nm23-H1-shRNA-luc细胞表达荧光素酶的稳定性

每次检测细胞生物发光值均设3个复孔，约隔周成像检测，共5次。其结果为：（3.14±0.55）×10^7^、（3.29±0.49）×10^7^、（3.27±0.25）×10^7^、（3.26±0.13）×10^7^、（3.12±0.22）×10^7^。各组相比差异无统计学意义（*P* > 0.05）（图 5）。这说明，随着细胞传代数的增加，细胞表达荧光素酶（luciferase）活性无明显变化，仍能维持在稳定水平。

**5:**
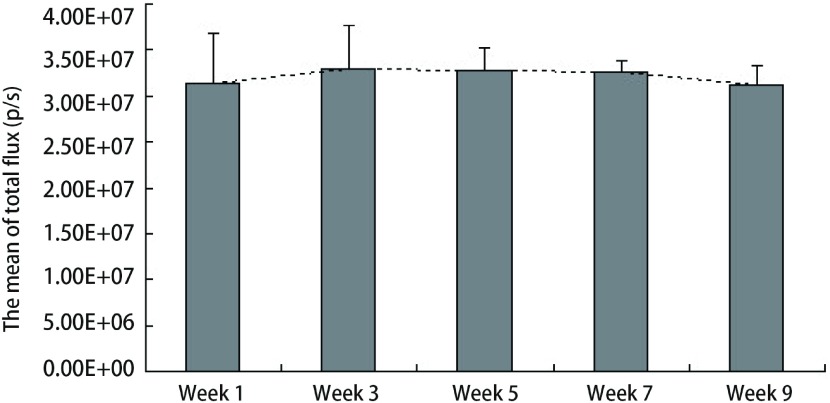
A549/nm23-H1-shRNA-luc细胞活体外生物发光值的稳定性评估示意图（*P* > 0.05） The bar chart to evaluate the stability of bioluminescence values of A549/nm23-H1-shRNA-luc cells *in vitro* (*P* > 0.05)

### 2.5 A549/nm23-H1-shRNA-luc细胞在裸鼠体内成像的观察及分析

A549/nm23-H1-shRNA-luc肺癌细胞株在裸鼠体内的荧光信号在接种后可以被检测到。随机把裸鼠种植瘤模型分成两组（5只/组），分别命名为：Group 1和Group 2（图 6），所测得的生物发光值均在同一数量级（10^9^）（图 7），两组所测得的生物发光值差异无统计学意义（*P* > 0.05）。

**6:**
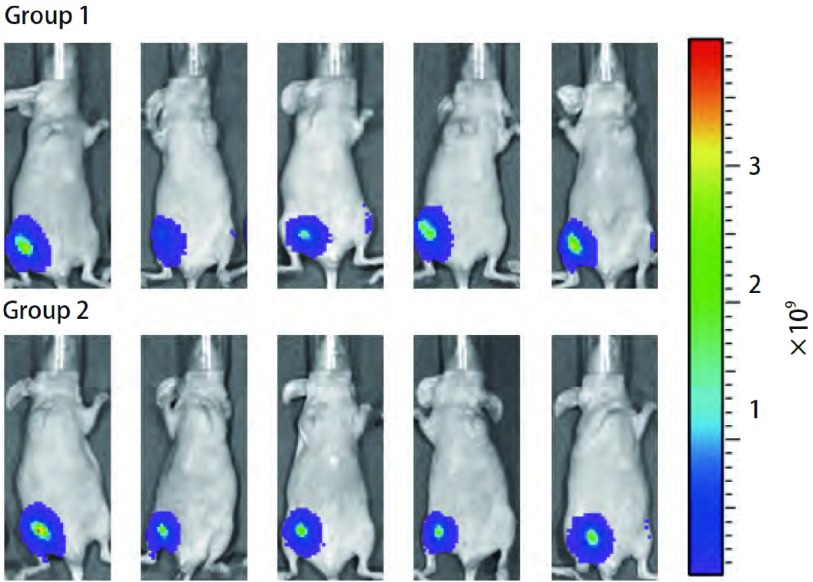
裸鼠种植A549/nm23-H1-shRNA-luc肿瘤细胞后活体成像图示 *In vivo* imaging of two groups of nude mice after A549/nm23-H1-shRNA-luc cells were implanted into nude mice

**7:**
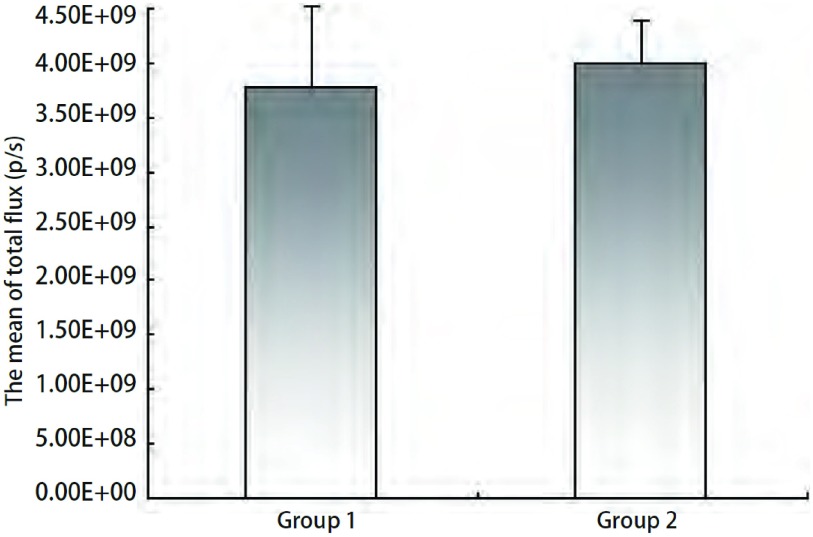
裸鼠种植A549/nm23-H1-shRNA-luc细胞后活体成像所测得的生物发光值 The bar chart of bioluminescence values *in vivo* after A549/nm23-H1-shRNA-luc cells were implanted into nude mice

## 3 讨论

随着对肿瘤分子生物学研究的深入，对基因、蛋白质功能的探索，我们最终研究的方向是在生物的活体环境中进行^[7-11]^。但传统的动物实验方法需要在不同时间点宰杀实验动物以获得数据，得到多个时间点的实验结果，其局限性主要表现为动物需求量大、实验过程中人为操作差异性大、实验过程耗时、获取信息量少等。近年来，随着微型非侵入性成像技术的发展，在实验动物环境中追踪肿瘤的发生发展成为可能^[12]^。相比之下，体内可见光成像通过对同一组实验对象在不同时间点进行记录，跟踪同一观察目标（标记细胞及基因）的移动及变化，由于能够对同一动物进行连续检测，在最大程度上减少了不同实验动物之间的个体差异以及传统检测方法的误差所造成的对实验结果的影响^[13]^。动物活体体内光学成像主要有生物发光（bioluminescence）与荧光（fluorescence）两种技术。生物发光技术是应用荧光素酶基因标记细胞或DNA，而荧光技术则采用荧光报告基团进行标记，然后利用灵敏的光学仪器直接检测活体生物体内的细胞活动和基因行为。生物发光技术相对于荧光技术而言，其具有检测的灵敏度高、特异性强、发光背景低、并能检测深部肿瘤等特点^[14]^，生物活体发光技术已成为当今体内研究的热点，其原理是在小的哺乳动物体内利用报告基因—荧光素酶基因表达所产生的荧光素酶蛋白（luciferase）与其小分子底物荧光素（luciferin）在氧，Mg^2+^存在的条件下消耗ATP发生氧化反应，将部分化学能转变为可见光能释放。因此只有在活细胞内才会产生发光现象，并且光的强度与标记细胞的数目线性相关。在体外利用敏感的CCD相机设备定量检测体内所发射的光子数量并将之转换成图像。它可以快速地测量各种癌症模型中肿瘤的生长，并可对癌症治疗中癌细胞的变化进行实时观测评估；可以无创伤地定量检测小鼠整体的原位瘤、转移瘤等^[15]^。其中，采用荧光素酶基因标记肿瘤细胞，使肿瘤细胞成为发光源，是后续建立移植瘤动物模型的基础。经过标记后的肿瘤细胞能够稳定表达荧光素酶，其发光强度和细胞的数量具有非常好的线性关系^[16]^。现行建立荧光素酶动物模型所使用的报告基因多为luc^+^型荧光素酶基因，本文中使用的是PGL4.50质粒载体携带有新一代luc2型高灵敏荧光素酶报告基因，其荧光素酶表达水平较普遍使用的luc^+^型有很大提高，催化底物荧光素发生氧化反应的效率更高，生物发光信号更强，因此对肺腺癌移植瘤动物模型中肿瘤细胞的检测更加灵敏。

结合本实验室的研究方向，我们希望构建出一个高转移肺癌移植瘤模型。本研究通过对nm23-H1表达缺失的人肺腺癌细胞株A549/nm23-H1-shRNA进行*luc*基因标记，构建出能在免疫缺陷的裸鼠体内生长、转移，并能稳定表达荧光素酶的细胞株A549/nm23-H1-shRNA-luc。本研究体外生物发光检测结果显示所筛选的稳定细胞株A549/nm23-H1-shRNA-luc的细胞数和其生物发光强度呈明显的线性相关，最小可探测到的细胞数为39个，且多次、间断的细胞体外活体成像数据显示，随着传代和时间的变化，该细胞株的生物发光值无明显改变（*P* > 0.05）（图 5）。体内生物发光结果显示，两组相同细胞在裸鼠体内所测得的生物发光值的差异无统计学意义（*P* > 0.05）（图 7）。以上体内外实验结果无一不证实了我们所构建的A549/nm23-H1-shRNA-luc细胞株是能够在非选择条件下稳定、长期地表达荧光素酶的细胞株。这种由活体成像技术所提供的数据，为以后判断肿瘤生长的大小提供了量化指标，从而为直接用生物发光强度判断肿瘤细胞在裸鼠体内生长和转移提供了依据。

下一步笔者将对建立好的nm23-H1表达缺失的肺腺癌移植瘤动物模型进行相关基因靶向治疗的研究。通过生物活体成像技术可以对治疗后动物体内肿瘤细胞进展变化进行量化以判断治疗效果^[17-20]^，这为基因治疗的研究及发展提供新的方向。
